# Data resource profile of an online database system for forensic mental health services

**DOI:** 10.1186/s12911-024-02433-2

**Published:** 2024-02-13

**Authors:** Junko Koike, Toshiaki Kono, Koji Takeda, Yuji Yamada, Chiyo Fujii, Naotsugu Hirabayashi

**Affiliations:** 1 Department of Community Mental Health and Law, National Institute of Mental Health, National Center of Neurology and Psychiatry, 4-1-1, Ogawahigashi, 187-8553 Kodaira, Tokyo, Japan; 2 Kawasaki City Inclusive Rehabilitation Center, 5-1, Nisshin-cho, Kawasaki Ward, 210-0024 Kawasaki, Kanagawa Japan; 3 Department of Forensic Psychiatry, National Center Hospital, National Center of Neurology and Psychiatry, 4-1-1, Ogawahigashi, 187-8551 Kodaira, Tokyo, Japan

## Abstract

This paper introduces a forensic psychiatry database established in Japan and discusses its significance and future issues. The purpose of this Database, created under the Medical Treatment and Supervision Act (MTSA) Database Project, is to improve the quality of forensic psychiatry treatment. It can collect monthly data on “basic information,” “Orders and hospitalizations under the MTSA,” “Treatment process,” “Criminal and medical treatment history,” and “problematic behavior in the unit.” The online system has accumulated data on more than 8,000 items in 24 broad categories. Medical data are exported from the medical care assisting system of 32 designated inpatient facilities in XML format and then saved on USB memory sticks. The files are imported into the Database system client, which sends the data to the Database server via a virtual private network. This system minimizes errors and efficiently imports patient data. However, there is a limitation that it is difficult to set items that need to be analyzed to solve everyday clinical problems into the database system because they tend to change over time. By evaluating the effectiveness of the Database, and collecting appropriate data, it is expected to disseminate a wide range of knowledge that will contribute to the future development of mental health and welfare care.

**Supplementary Information:**

The online version contains supplementary material available at 10.1186/s12911-024-02433-2.

## Introduction

In the era of evidence-based treatment and policymaking, administrative data provide essential information on health service utilization, expenditures, and clinical outcomes, which is necessary for assessing the quality of care (Chowdhury et al., 2015). In forensic psychiatry, some previous studies have pointed to the effectiveness of administrative data utilization [1]. However, surveys are generally based on medical data, including electronic medical records from each medical institution or even registry data [2].

Forensic psychiatry plays a pivotal role in the public health crisis of the mentally ill, and MTSA plays a role in the provision of medical care to mentally ill offenders in Japan. Under the Medical Treatment and Supervision Act (MTSA), the MTSA database system specific to forensic psychiatry has been developed, involving the constant collection of data from 35 forensic psychiatric hospitals. In addition, rules and procedures for the secondary use of the stored data for research purposes have been developed, and organizational structures such as a steering committee and a review committee have been established. This paper introduces the Database system and discusses the significance of the Database and future issues.

### Overview of legislation pertaining to forensic mental health services

The Japanese system for treating mentally disordered offenders is introduced here, along with an overview of the MTSA as a forensic psychiatric care system.

Under Japanese law, article 39 of the Penal Code stipulates the following:

1. An act under insanity is not punishable.
2. An act under diminished capacity shall lead to the punishment being reduced.

In Japan, medical care is provided to mentally disordered offenders at a separate setting for each of the points listed below, and each location has its own legal requirements and treatment methodologies.

1. No definition for Unfitness/Incompetence to Stand Trial exists in Japan. Medical care is provided in medical facilities operated in the criminal justice system when people need psychiatric medical treatment after having entered a correctional facility (The Act on Penal Detention Facilities and Treatment of Inmates and Detainees, Article 56).
2. The MTSA concerns individuals who have committed serious harm while insane. Under this Act, the District Court makes decisions regarding treating certain mentally disordered offenders. This Act aims to prevent recidivism that can harm others and to encourage resocialization.
3. Individuals with mental disorders who commit self-harm and/or harm others, excluding serious harm to others, are involuntarily admitted to receiving psychiatric treatment. This medical intervention is forced under the Mental Health and Welfare Act (Article 29).

Since the details and features of the system associated with the MTSA have already been published [3, 4], only a brief overview is given here. When the “referral requirement” of the MTSA is satisfied, the public prosecutor must make a referral to the district court (Article 33, Paragraph 1). This Act applies to people who have committed serious harm (such as homicide, arson, robbery, rape, indecent assault, or injury) and have been put to no prosecution, sentenced not guilty, or given a suspended sentence. If the prosecutor referred the person to MTSA process (Article 33), in this case, a panel consisting of a judge at the District Court and an attending psychiatrist decides whether a treatment order under the MTSA can be applied (Article 42). The District Court shall order an evaluation by a specially qualified psychiatrist regarding a determination of the existence of a mental disorder and an opinion on the necessity of medical treatment under this Act. (Article 37).

Following these procedures, if the panel judged that treatment under the MTSA is necessary to improve the referred person’s mental condition and aid in social reintegration without reoffending, the District Court can order the individual to undergo either inpatient or outpatient treatment order, or no treatment order according to the MTSA. In the case of inpatient treatment order, the subject is treated in the forensic unit of a designated inpatient facility. In the case of outpatient treatment order, the subject is treated at a designated outpatient facility. Most subjects who have received inpatient treatment may receive outpatient treatment after discharge from a designated inpatient facility. If the patient’s mental condition worsens during outpatient treatment, they will be readmitted to designated inpatient facility. If the patient violates any of the requirements for outpatient treatment or fails to participate in a mandatory interview with a social rehabilitation coordinator, they will also be readmitted to a designated inpatient facility and receive psychiatric treatment (Article 61, Paragraph 1). After completing outpatient treatment, many patients are transferred to general psychiatric services for further treatment.

The length of treatment at a designated inpatient facility is not specified by the MTSA. However, according to treatment guidelines issued by the government, the period for treatment in a designated inpatient facility is 1.5 years. And outpatient treatment is 3 years (with a maximum possible extension of 2 years) (Article 44).

The designation and provision of treatment facilities are as follows. The national government designates inpatient and outpatient facilities. Only national or prefectural hospitals may be designated inpatient facilities. There are no restrictions on the provider of designated outpatient facilities. As of April 1, 2022, there are 35 designated inpatient facilities and 676 designated outpatient facilities (589 hospitals and 87 clinics) nationwide. Including 2,608 pharmacies and 570 home-visit nursing stations, there are a total of 3,854 designated outpatient facilities.

### Background of MTSA database development

Immediately after the MTSA went into effect, there was no systematic monitoring system to evaluate the operation of the MTSA. Public research funds supported research on the actual condition of treatment, but there were challenges in continuity and thoroughness. Because of the significant public funding for the MTSA and involuntary treatment programs, statistical data were needed to explain the reality and outcome of treatment and practice. Additionally, the MTSA was expected to improve the quality of general psychiatric care by monitoring and evaluating treatments and procedures of the MTSA. Therefore, a database was needed to collect and analyze all patient data and contribute to clinical practice.

In July 2017, the MTSA Database Project (translated official name: “Project for Establishment of Standard Treatment for Severe Mental Disorders”) was initiated. In October 2022, 32 of 35 designated inpatient facilities are submitting data. Data on all inpatients will be collected as soon as the remaining 3 designated inpatient facilities complete the necessary procedures. Between July 15, 2005, and March 2022, data on more than 4,000 cases have been accumulated, and those of approximately 250 additional cases are expected to be added annually.

### MTSA database project

The primary purpose of the MTSA Database Project is to collect and analyze data on inpatient care under the MTSA. It also aims to provide feedback to designated inpatient facilities, thereby contributing to the appropriate implementation and improvement of the standard of medical care and to the promotion of the resocialization of subjects of the MTSA. Finally, this project aims to collect and analyze medical information and provide feedback to designated inpatient facilities. The Ministry of Health, Labour, and Welfare (MHLW) is paying for this project, and it is being implemented by 35 designated inpatient facilities, including the NCNP Hospital, which serves as the chief hospital. The steering committee comprises about 10 members, including psychiatrists based at the aforementioned facilities and experts in psychiatry and jurisprudence. Based on the draft prepared by the working members of the Steering Committee, the committee decides on matters related to the project’s operation.

The data to be collected in the Database consists of 24 minor categories, which can be broadly divided into “basic information,” “Orders and hospitalizations under the MTSA,” “Treatment process,” “Criminal and medical treatment history,” and “problematic behavior in the unit.” Each of the 24 major categories has broad categories, and more than 8,000 data items have been collected. Major categories and Broad categories of collected data in the Database were showed supplementary Appendix 1.

**Fig. 1:**
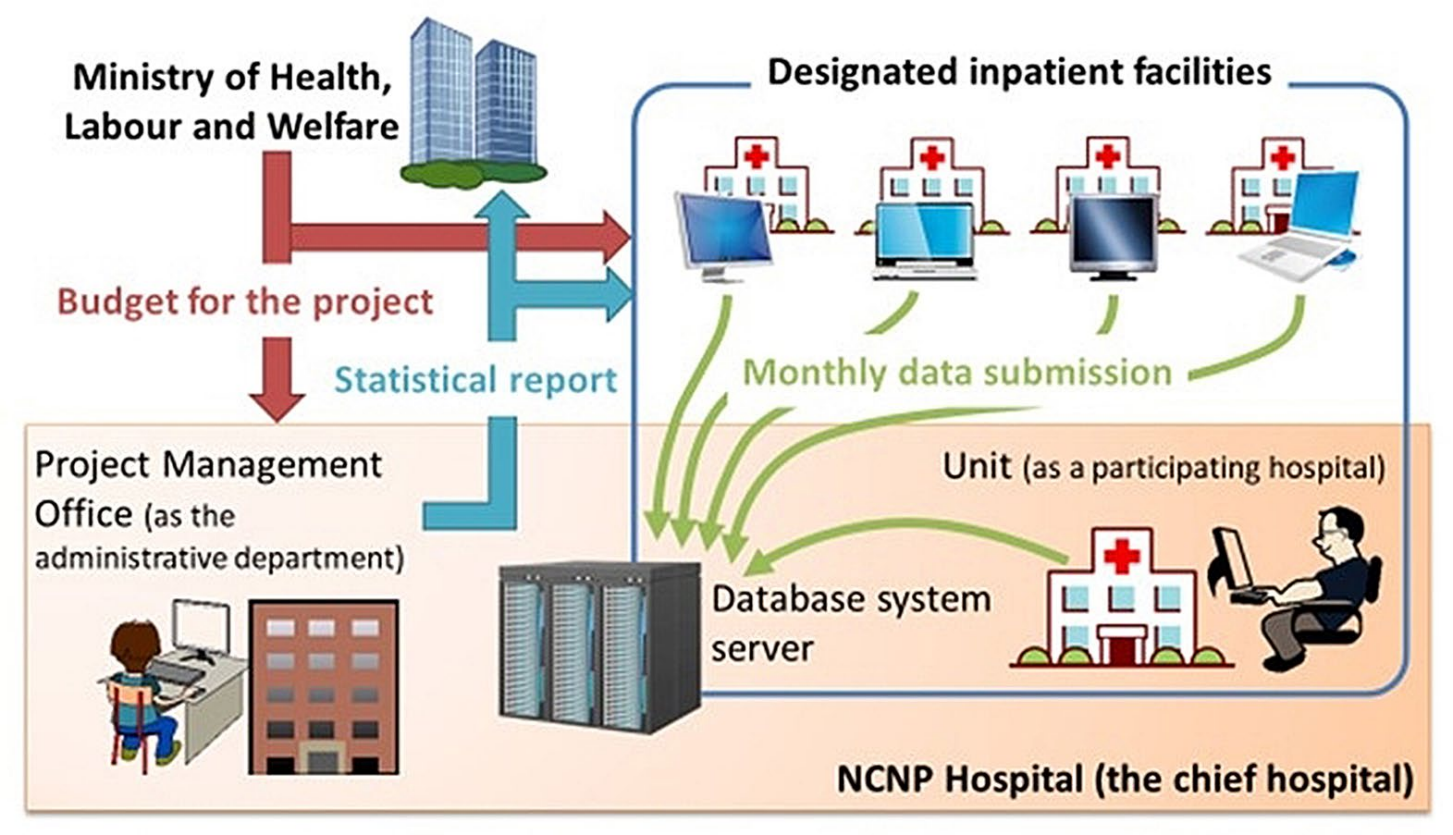
Overview of the MTSA database project. In the project, the Ministry of Health, Labour and Welfare expends the budget for the project to NCNP hospital, the chief hospital of this project, as well as the other designated inpatient facility. The facilities submit data on medical services once a month via a newly developed online system to a server equipped in NCNP hospital. Analyses are performed in Forensic Psychiatry Clinical Research Center and statistical reports are fed back to the ministry and the hospitals

**Fig. 2:**
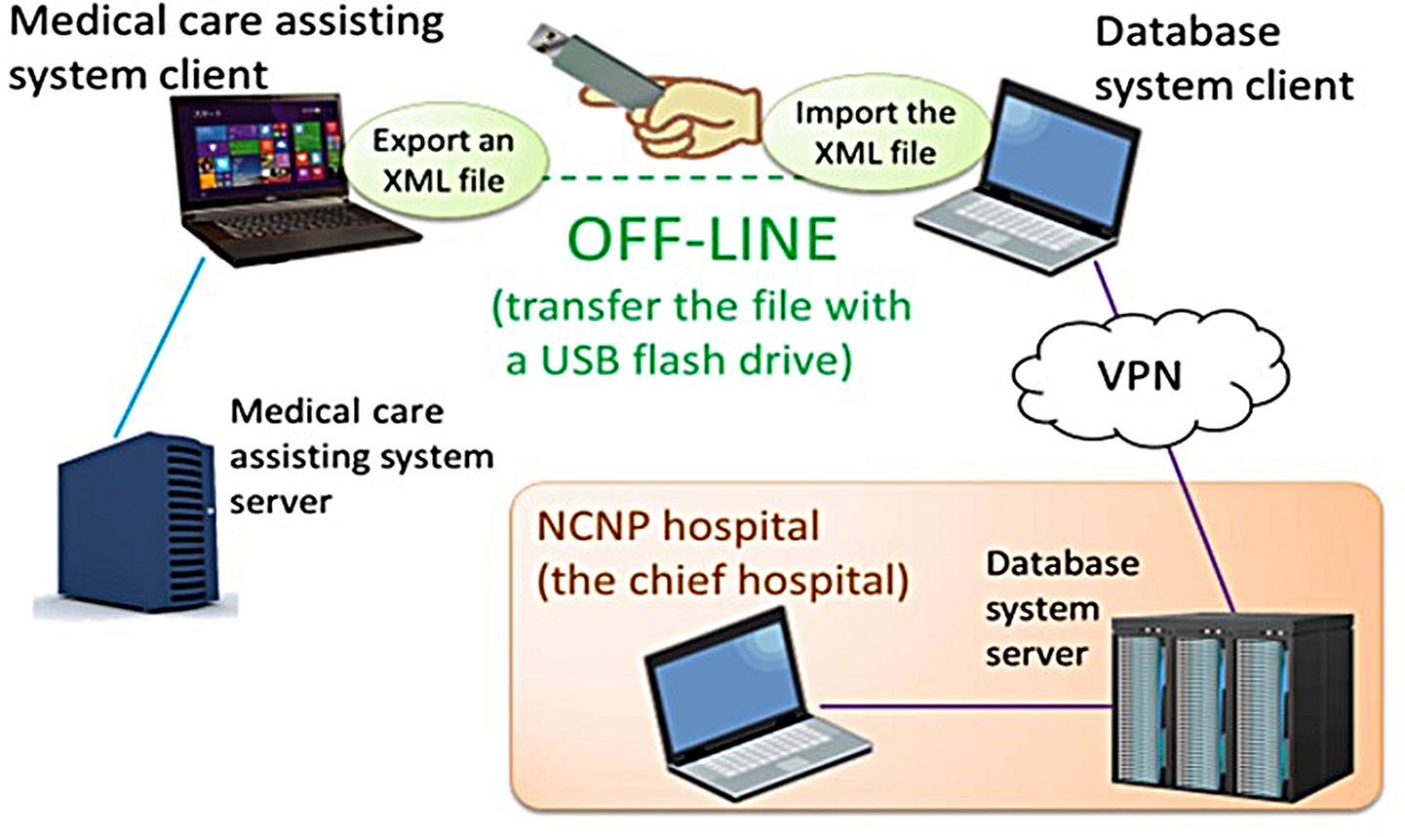
All the collected data are existing information input to so-called medical care assisting system which is an electronic medical chart system specially developed for the MTSA secure units. The medical staff doesn’t need to input any additional information only for database. A staff export data to submit as a file in XML format from the medical care assisting system and save the file in a USB flash drive. Then the staff import the file to the database system and send the data via a virtual private network to the server

All data collected comprise existing information that is entered into an electronic medical record system (medical care assisting system) exclusively developed for the MTSA. The medical staff does not need to collect and enter additional data for the MTSA Database Project; they submit their medical data to the server of the NCNP hospital (the chief hospital) at monthly intervals through the developed online system (Fig. 1). The medical care assisting system exports the data to be submitted in XML files that are stored on a USB memory stick. The files are then imported into the Database system client, which sends the data to the Database system server via a virtual private network (Fig. 2).

The transmitted data are treated as personal information. Personal information cannot be collected without the patient’s consent, but as an exception, laws and ordinances related to the protection of personal information allow the use of such information when necessary and appropriate to fulfill legal obligations or to prepare statistical data (Revised Personal Information Protection Law). In this system, collected data are anonymized and stored with an identification number used in the project. The Database system has made it possible to perform error checking, ensure thorough data correction procedures, and manage data more efficiently.

The data obtained from this project are analyzed by the NCNP hospital as care performance indices (6-monthly reports) and reported to the MHLW. The main items reported are “age,” “diagnosis,” “index offense,” “stage of treatment,” “length of hospital stay,” “behavioral restrictions,” “modified electroconvulsive therapy,” “pharmacotherapy,” and “outcome.” The NCNP hospital provide feedback on these results to designated inpatient facilities to promote the appropriate clinical application and standardization of the quality of care. Each designated inpatient facility can conduct analyses using its registered data and medical evaluations within the hospital.

### The MTSA database scientific utilization project

A framework related to the MTSA Database Scientific Utilization Project [5] was established in October 2020 to allow secondary use of the collected data for research. This service was part of a research project conducted by the National Center of Neurology and Psychiatry. The scope of use in this project is as follows.

- Scope of data that can be provided. All medical information is registered in the MTSA Database (however, at the time of provision, the data will be processed so that individuals cannot be identified).
- Requirements regarding the purpose of use. The research must contribute to improving the standard of psychiatric care, etc.
- Requirements for applicants/users. Only employees of designated inpatient facilities may submit applications. All other people (researchers, etc.) must use the data under the supervision of the applicant.

In October 2022, information on more than 4,000 inpatients was collected in the Database. Additional information on approximately 250 inpatients is expected to be added annually. Currently, there are six applications for secondary use research, and five of the six applications have already been approved.

### Characterization of people with mental disorders under the MTSA using database data

This section presents the MTSA Database Project data on 3,761 individuals who began to receive inpatient treatment between July 15, 2005, and December 31, 2020. The results of this tabulation of the data were published in the 2020 edition as the first administrative data on persons subject to the MTSA [6]. The following data was quoted from ‘ The Report of Medical Treatment and Supervision Act 2020´ published by the Steering Committee.

1. Age (Fig. 3). Patients in their thirties and forties accounted for the highest percentage overall. The average age at admission tended to become higher after the MTSA was first enacted but has leveled off since around 2012.
  **Fig. 3:**
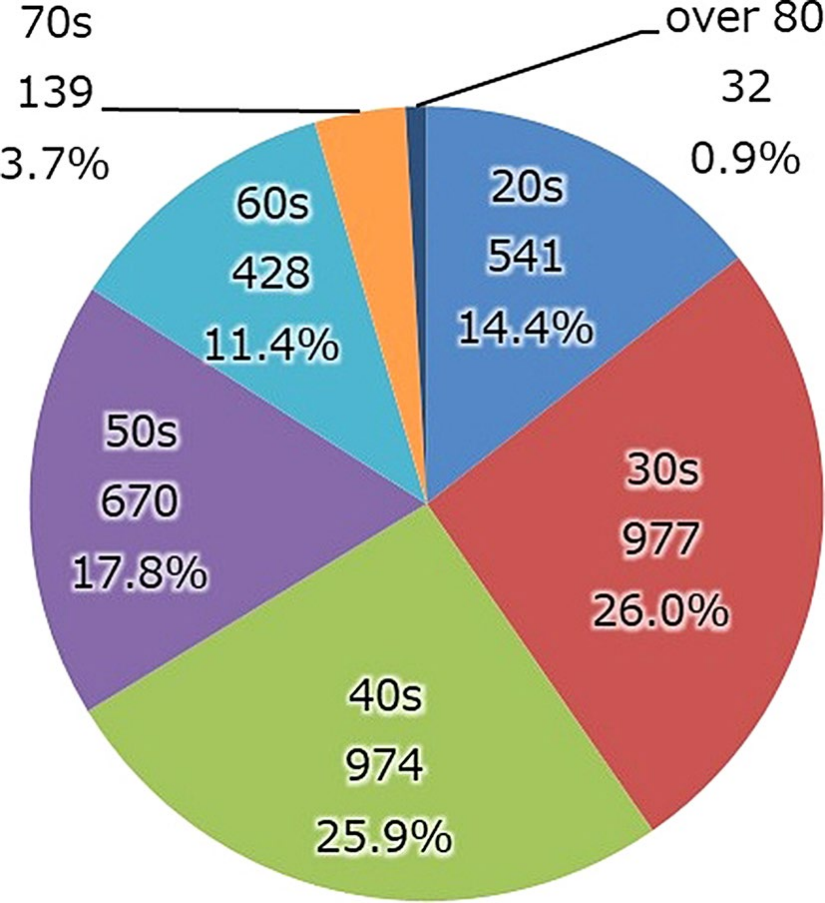
Patients in their 30 and 40 s account for half of all inpatients
2. Gender (Fig. 4). The male-to-female ratio in the cumulative data is approximately 3:1 and has not changed significantly over time.
  **Fig. 4:**
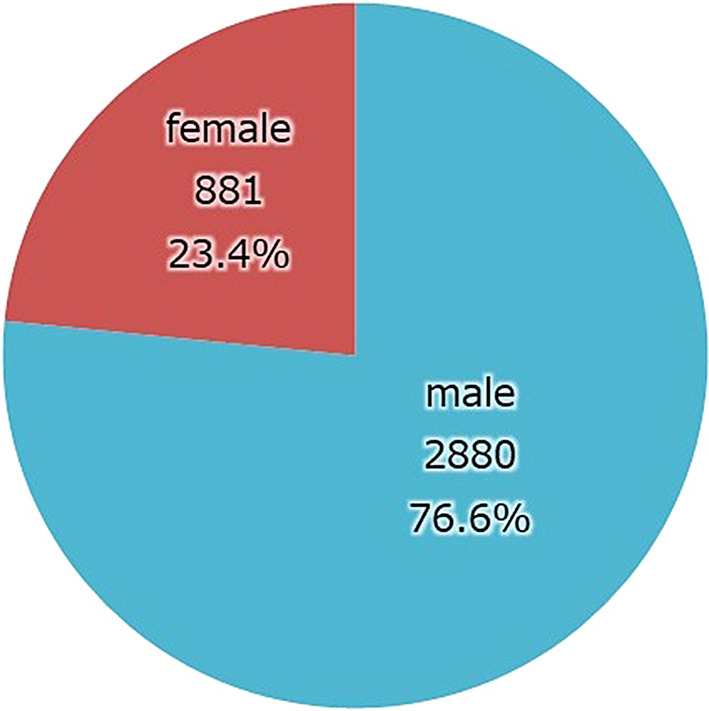
The percentage of male inpatients is 76%, while the percentage of female inpatients is 23%
3. Principal diagnosis (Fig. 5). This Database is the 10th revision of the International statistical classification of diseases and related health problems (ICD-10) compliant. F2 accounted for about 80% of the primary psychiatric diagnoses, followed by F3 and F1.
  **Fig. 5:**
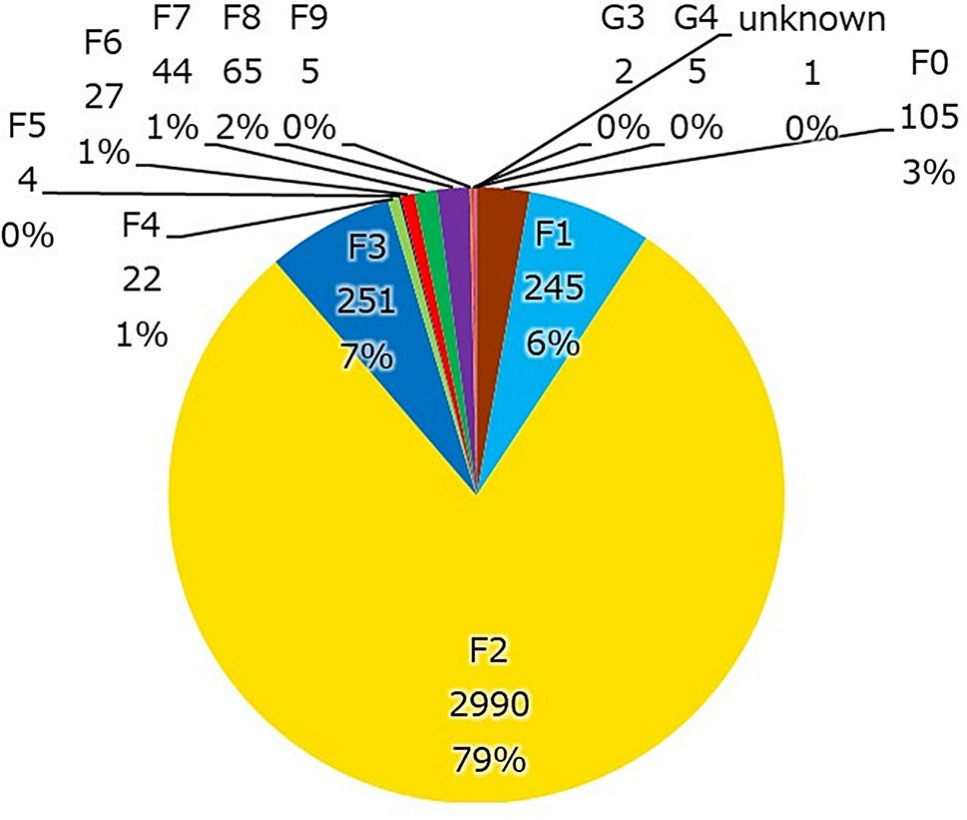
F2 is the most frequent diagnosis of psychiatric disorders
4. Co-morbid disorders (Fig. 6). The most common co-morbid disorders are F7, F1, and F8.
  **Fig. 6:**
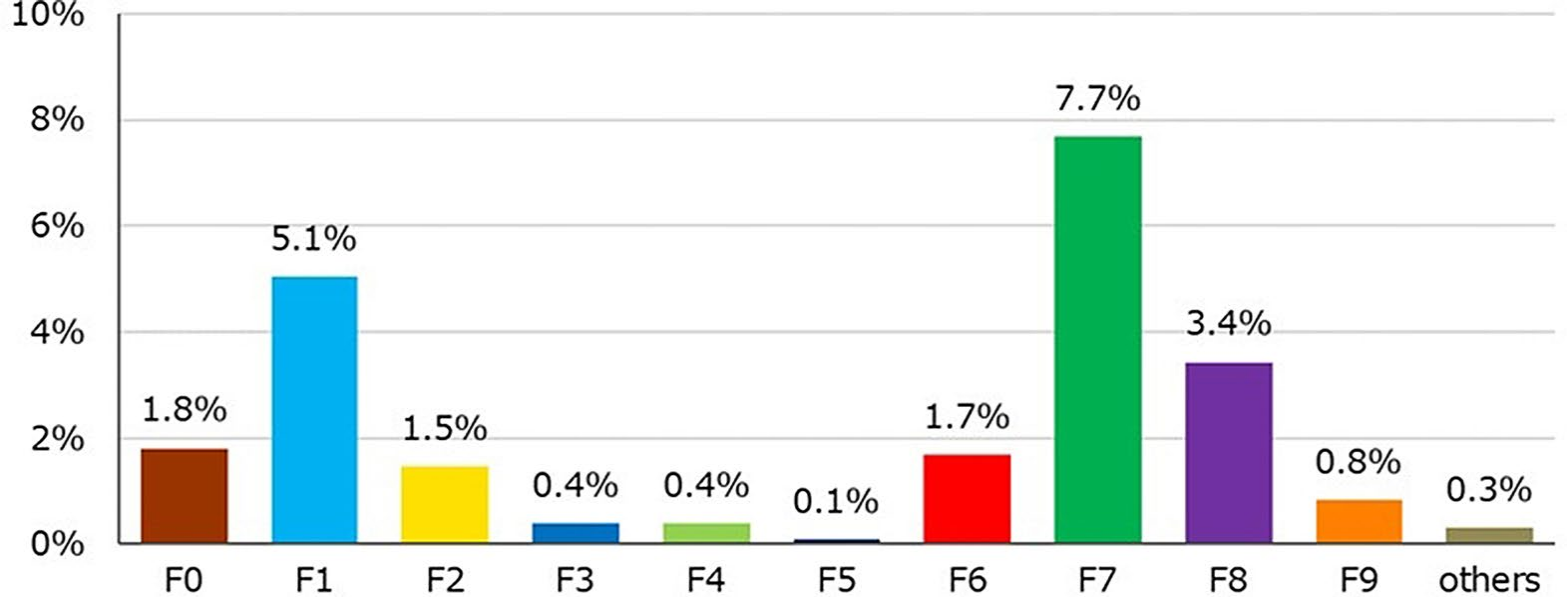
This database can collect up to two comorbid disorders; F7 has the highest rate
5. Index offense (Fig. 7). The primary index offense was counted and included attempted offenses. The most common were injury, followed by homicide, and arson; robbery, rape, and indecent assault were less common.
  **Fig. 7:**
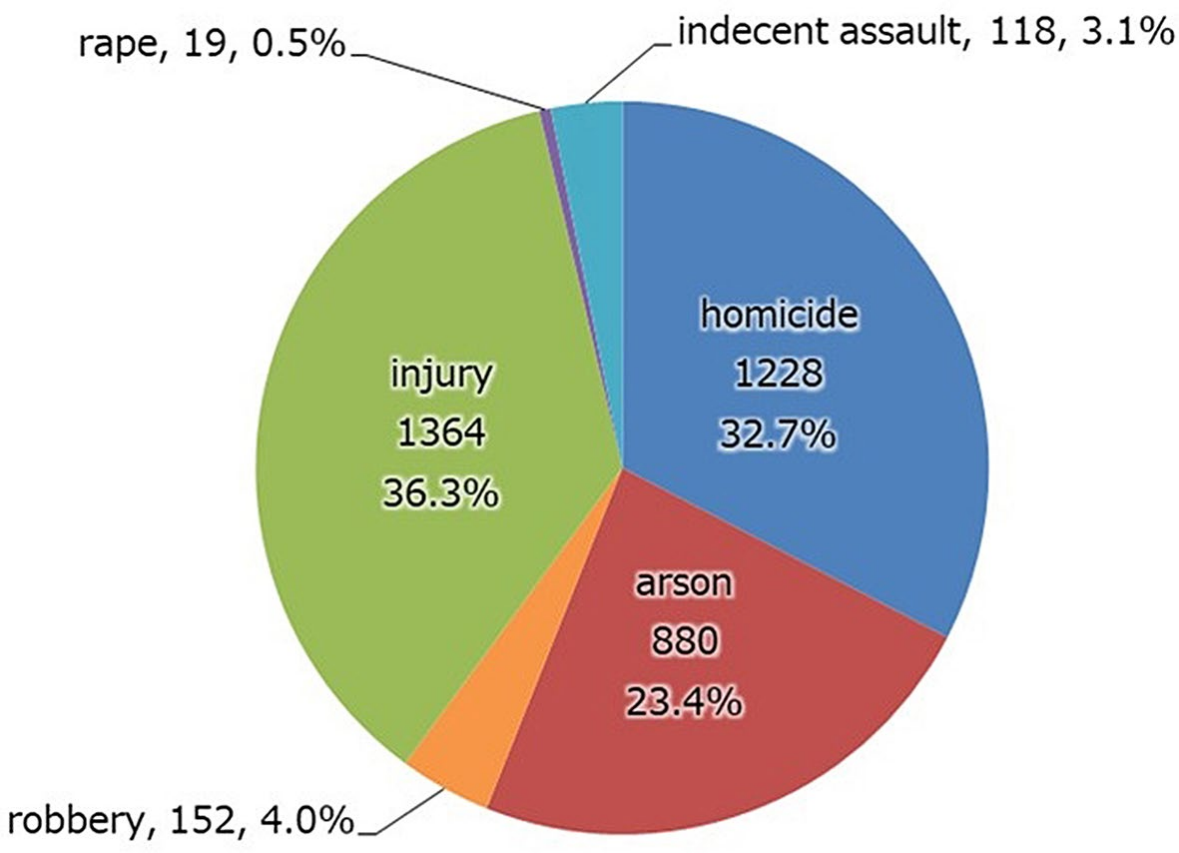
Index crimes include attempts. Injury, homicide, and arson account for more than 90% of the total number of crimes
6. Outcome (Fig. 8). About 80% of the discharged subjects were transferred to outpatient treatment.
  **Fig. 8:**
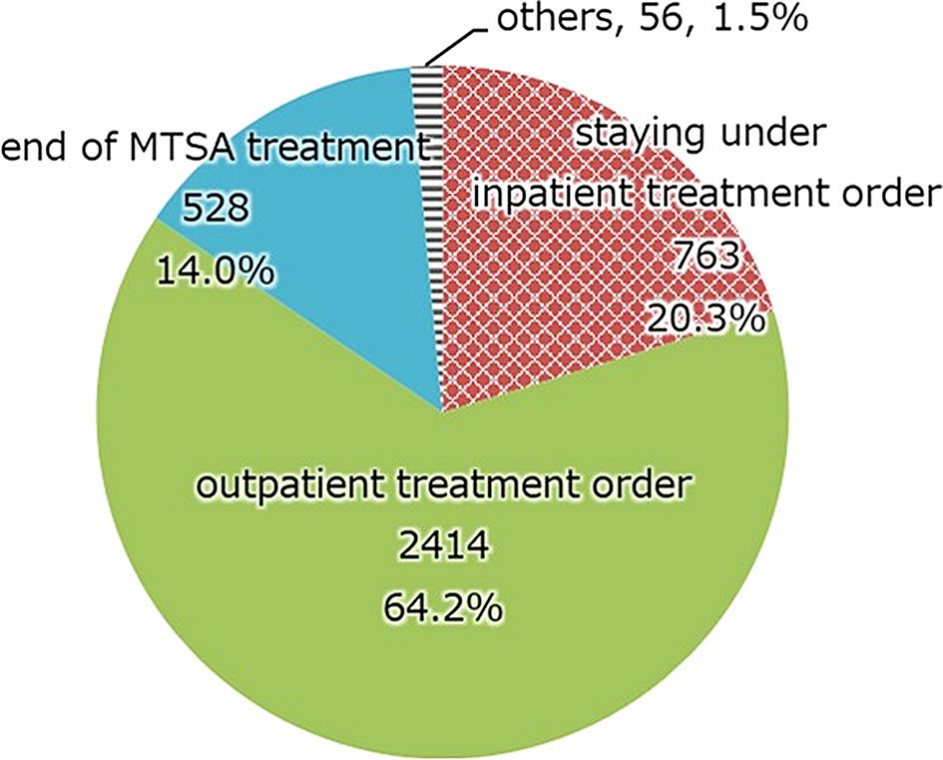
This figure shows the percentage of treatment status at one point in time. 60% of those who committed serious harm to others are in outpatient treatment, and about 80% of discharge subjects are receiving community support under the MTSA
7. Length of hospital stay (Fig. 9). The cumulative length of hospital stay was analyzed using the Kaplan-Meier method. The average length of hospital stay was 1,022 days, and the median length was 827 days. About 20% of the patients were in a designated inpatient facility for 3–5 years, and about 10% were in such a facility for more than 5 years.
  **Fig. 9:**
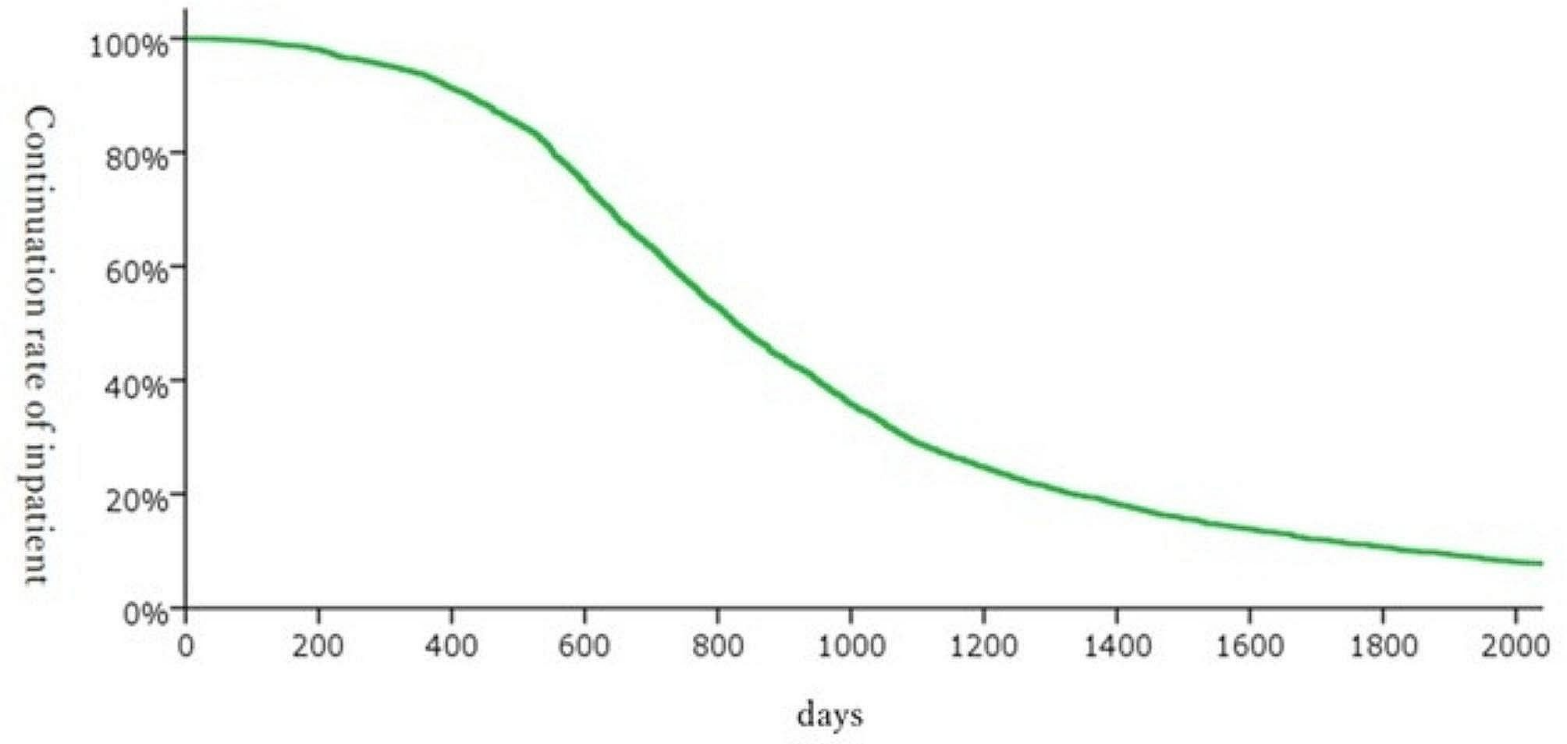
Between 600 and 1,200 days, about 50% of inpatients were discharged from the designated inpatient facilities; about 30% of patients stayed in the facilities for more than 3 years

Also presented below are the results of previous studies that conducted statistical analyses using data from The MTSA Database Scientific Utilization Project.

Kono et al. classified inpatients under the MTSA using TwoStep cluster method [7]. A total of 28 clusters were generated. Restricting to those with a principal diagnosis of F2, 9 clusters were extracted; 4 clusters were defined by co-morbid disorders, while the remaining 5 were defined by age and gender. Among them, very long hospitalizations (5 years or longer) were more frequent in younger patients, in patients with co-morbid F8, and in female patients with co-morbid F7. In addition, Kabeya analyzed the segmentation of groups that had to be long-term seclusion, restraints, and hospitalization [8].

Koike et al. examined the relationship between length of hospitalization and patients’ basic characteristics [9]. In the relation between length of hospitalization and age, younger and elderly inpatients had higher rates of short-term hospitalization. Concerning principal diagnosis, F0, F1, and F7 tended to have shorter hospital stays, while F2 showed a trend toward fewer short-term hospitalizations (𝛘^2^ = 245.869, *P* < 0.001). Concerning co-morbid disorders, F0 showed a trend toward shorter hospitalizations (𝛘^2^ = 16.32, *P* < 0.001), and F8 showed a trend toward more extended hospitalizations (𝛘^2^ = 43.715, *P* < 0.001). Inpatients who committed homicide were less likely to have a hospital stay of 2 years or less (𝛘^2^ = 22.178, *P* < 0.001).

## Discussion

### Significance of the database and current issues

The database system enables the monthly collection of approximately 8,000 items of data regarding subjects admitted to 35 designated inpatient facilities under the MTSA. In forensic psychiatry, it has been noted that basic national epidemiological data are lacking, and factors associated with recidivism prevention have not been adequately identified [10–12]. To resolve this problem, demographic data, clinical data, and data on static and dynamic factors related to crime have been collected using databases in Sweden [13] and Germany [12]. The items collected do not differ significantly between Japan and the other countries. The Japanese database is designed for administrative rather than research purposes and is unique in that all patient data can be collected over a long period, but it is not suitable for effectiveness evaluation.

The method data is collected, that it is not necessary to re-enter routine clinical records to accumulate data in the Database is particularly beneficial from the perspective of efficiently collecting data that is also clinically necessary. The Database is used in several ways:

- Monitoring of appropriate healthcare provision by healthcare professionals.
- Improvement of the quality of medical care (self-institutional review, institution-to-institution advice).
- Discovery of the need for new treatment techniques, etc.
- Identification of issues to establish a comprehensive community support system.

On the other hand, this Database system is associated with challenges related to the following issues:

- Regular publication of administrative data.
- Promotion of research use.
- Consideration of Database items.

In 2022, the first statistical document was published. It is hoped that this document will be published regularly, and to this end it is necessary to establish a publication system. In addition, only five secondary-use research have been approved in about 2 years. Therefore, measures to promote research use are needed.

In consideration of the items in the Database, what should be noted is the historical circumstances of Japan’s treatment system for persons with mentally disordered offenders, as well as the development of the appropriate application of the forensic system. Before the MTSA was enacted in Japan, general psychiatric hospitals provided treatment for mentally disordered offenders. Several issues arose regarding psychiatric treatment: (1) human rights issues (security measures and detention without treatment) [14, 15], (2) disadvantages of mental health policy assuming responsibility for social defense [16], (3) problems with the length of hospitalization (hospitalizations tend to be extremely short or long) [17–20], (4) lack of continuity of medical care [21], (5) lack of human resources and medical fees [22], and (6) the fact that a single doctor makes the decision to terminate involuntary hospitalization without the need for a judicial decision [23].

Not all issues have been resolved with the enforcement of the MTSA. However, in light of these historical challenges, the Database focuses on comprehensive and continuous monitoring of ethical aspects such as compulsory medical care and designing an appropriate healthcare system. Therefore, one anticipated role of the Database is to enable comprehensive and continuous monitoring of ethical issues. In this sense, the Database is helpful as a method of ascertaining the operation of the MTSA regarding subjects, treatments, and lengths of hospital stay. In fact, the average hospital stay is 1,022 days, the median stay is 827 days, and 90% of patients are discharged within 5 years. In addition to advice from the Ministry of Health, Labor, and Welfare, such a short hospital stay has been achieved through the self-help efforts of designated inpatient facilities. The Database provides the basis for these medical activities.

As previously described, the Database captures items that reflect the appropriateness of the appropriate application of the system. On the other hand, healthcare providers want to be able to capture static and dynamic factors data on the variety and effectiveness of the treatment being provided, factors related to discharge, and other data to help solve routine clinical issues [24]. Tomlin et al. [12] also emphasized the necessity of comprehending data of both static and dynamic factors concerning cost, recidivism prediction, and treatment outcomes within forensic psychiatry databases. However, it has been reported that the collection of dynamic data within these databases is not without its challenges. In Germany, issues such as the irregular collection of dynamic data in everyday practice, the risk of data errors during the data collection process, and the influence of researcher presence have been cited [12]. In Japan, there is no need to re-enter clinical records if the dynamic factor data aligns with metrics routinely evaluated in clinical settings. Nevertheless, challenges similar to those encountered in Germany may arise when attempting to accommodate the collection of additional data items and the needs of staff. Collecting dynamic data within a database appears to present a significant challenge.

The database items are expected to change with time and circumstances. If the Database item is modified based on the needs of healthcare providers, it will be necessary to evaluate the appropriateness of the selection process of database items. It will be expensive to modify the Database system each time the composition of collected data items is changed. In response to this, it will be necessary to verify that all users understand the Database. In addition, without a cooperative relationship with healthcare professionals, it will be difficult to promptly correct input errors and to consider the scope of these professionals’ effective use of large amounts of data. Evaluating the Database Projectthat can be disseminated to the world is a future task.

### Prospects for treatment of mentally disordered offenders using the database

Although there are still significant issues to be addressed in the Database Project, we believe it will facilitate the assessment of forensic psychiatry and the overall treatment system for persons with mental disorders, including treatment in general mental health services and prisons.

As noted in previous studies, mentally disordered offenders tend to have ongoing difficulties due to multiple issues in life that began in childhood [25]. It has also been reported that the development of psychotic disorders is increasingly associated with violence [26]. In particular, factors related to recidivism include psychotic disorders, minimal education [27, 28], and illicit substance use from an early age [29]. In the case of women, there is a strong association with past experiences of abuse [30, 31]. In Japanese studies, mental disorders are often the result of challenging upbringings and background factors in life, suggesting that repetition of harm to others occurs before the onset of mental disorders [24, 32]. Reports from prisons also point out a high prevalence of psychiatric disorders, especially among low- and middle-income inmates [33] and a high proportion of mentally disabled inmates [34].

Analysis of data from the database showed a relationship between co-morbid disorders and length of hospital stay. Inpatients with co-morbid disorders may be complex cases requiring long-term support. For such inpatients, it is necessary to clarify the scope of application of the MTSA, improve support techniques, and develop community mental health systems. In forensic psychiatry, there is no unified view on the appropriate length of hospitalization and effectiveness of treatment for mentally disordered offenders with intellectual and developmental disabilities [35], therefore accumulation of knowledge is required.

Analysis of the database data will make it possible to identify the psychopathology and life obstacles of mentally disordered offenders and thus provide practical knowledge for ex post facto medical and welfare support measures. By evaluating the effectiveness of the database, and collecting appropriate data, it is expected to disseminate a wide range of knowledge that will contribute to the future development of mental health and welfare care.

### Limitation

We consider that this database is large in scale as one for mentally disordered offenders. However, it has several limitations. First, it does not collect enough assessment items to meet clinical needs. Second, this database only collects data on inpatient treatment and does not collect data on outpatient treatment. We intend to develop the database further to make it more useful.

## Conclusion

The purpose of this paper was to introduce a forensic psychiatry Database in Japan and to discuss its significance and future issues. This Database can be used to obtain information on forensic inpatient treatment in Japan, as related to diagnoses, index offenses, psychiatric symptoms, risk of harm, length of hospital stay, and outcomes. On the other hand, there are limitations in ascertaining indicators that change over time, as well as variables that contribute to resolving clinical issues. By evaluating the effectiveness of the Database, and collecting appropriate data, it is expected to disseminate a wide range of knowledge that will contribute to the future development of mental health and welfare care.

### Electronic supplementary material

Below is the link to the electronic supplementary material.

Supplementary Material 1

## Data Availability

Existing summary data were cited in this paper. For this reason, the raw data are not available. Summary data shown in ‘The Report of Medical Treatment and Supervision Act 2020’ are available from the National Center of Neurology and Psychiatry (NCNP) website (only in Japanese).

## References

[1] Kleinpeter C, Deschenes EP, Blanks J, Lepage C, Knox M. Providing recovery services for offenders with Co-occurring disorders. J Dual Diagnosis. 2006;3(1). 10.l300/j374v03n01_06.

[2] Noland E, Strandh MH. Clinical and situational risk factors for post-discharge recidivism in forensic psychiatric patients - a Swedish registry study. Int J Law 2021 Psychiatry, 79, 101749. 10.1016/j.ijlp.2021.101749. Epub 2021 November 10. 10.1016/j.ijlp.2021.101749 34768026

[3] Nakatani Y, Kojimoto M, Matsubara S, Takayanagi (2010). I New legislation for offenders with mental disorders in Japan. Int J Law Psychiatry.

[4] Fujii C, Fukuda Y, Ando K, Kikuchi A, Okada T (2014). Development of forensic mental health services in Japan: working towards the reintegration of offenders with mental disorders. Int J Mental Health Syst.

[5] The MTSA Database Scientific Utilization Project. [https://www.ncnp.go.jp/hospital/patient/mtsa.html]. Accessed 12/06/2023.

[6] MTSA inpatients report. [https://www.ncnp.go.jp/shiryou/iryokansatsuho.html]. Accessed 12/06/2023.

[7] Kono T, Koike J, Takeda K, Kabeya Y, Soshi T, Okano M, Fujii C (2023). Hirabayashi, N. exploratory typification of under the inpatients under the Medical Treatment Supervision Act. PSYCHIIATRY.

[8] Kabeya T (2023). Analyses on prolonged hospitalization frequent/long behavioral restrictions, and termination of treatment under the Medical Treatment Supervision Act. PSYCHIIATRY.

[9] Koike J, Kono T, Okano M, Takeda K, Fujii C, Hirabayashi N (2023). Factors associated with length of hospitalization for inpatients under the Medical Treatment Supervision Act. PSYCHIIATRY.

[10] Tomlin J, Lega I, Braun P, Kennedy HG, Herrando VT, Barroso R, Castelletti L, Mirabella F, Scarpa F, Völlm B (2021). Forensic mental health in Europe: some key figures. Soc Psychiatry Psychiatr Epidemiol.

[11] Fovet T, Saint-Dizier C, Wathelet M, Horn M, Thomas P, Guillin O, Coldefy M, D’Hondt F, Amad A, Lamer A (2023). Opening the black box of hospitalizations in French high-secure psychiatric forensic units. Encephale.

[12] Tomlin J, Walde P, Völlm、B. Protocol for the CONNECT Study: A National Database and Prospective Follow-Up Study of Forensic Mental Health Patients in Germany. Front. Psychiatry, 25 April 2022 Sec. Forensic Psychiatry. Volume 13–2022, 10.3389/fpsyt.2022.827272. 10.3389/fpsyt.2022.827272 PMC9081526 35546932

[13] Sivak L, Forsman J, Masterman T. Duration of forensic psychiatric care and subsequent criminal recidivism in individuals sentenced in Sweden between 2009 and 2019. Front Psychiatry. 2023;14(1301759). 10.3389/fpsyt.2023.1129993. eCollection 2023. 10.3389/fpsyt.2023.1129993 PMC10053040 37009123

[14] Asada K (1982). Procedures concerning persons with Mental Disordered offenders under the Law and the Role of Psychiatric examinations. Monthly Jurist.

[15] Kusumoto T (2002). A Summary of the Security Disposition Debate. Houritujihou.

[16] Nakatani Y, Matsushita M (1998). Crime and Mental Health Administration: Post war Trends. Clinical Psychiatry Course 19: J*udicial Psychiatry and Psychoanalysis*.

[17] Yamagami A, Konishi T, Yoshikawa K, Inoue T, Sha R (1995). An 11-Year follow-up study of 946 mentally disordered offenders. (1st report). Outline of 487 crimes committed by 207 offenders. Acta Criminologiae et medicinae lagalis Japonica.

[18] Nakatani Y (1996). Community mental health and mentally disordered offenders. Seishinkatiryougaku.

[19] Tsukue I, Konuma (2002). K result of urgent investigation into the actual condition of serious criminal patients hospitalized after psychiatric evaluation in conformity with the mental health welfare law. Seishinigakuzasshi.

[20] Tsukue I, Konuma K (2002). Consideration of measures for mentally disordered offenders. Who have committed serious crimes and have been prosecuted. J Japanese Association Psychiatric Hosp.

[21] Nagao T (1998). Problems with the system of hospitalization for mentally disordered offenders. J Japanese Association Psychiatric Hosp.

[22] Takei M (2003). Health Care Economic structure of Psychiatric hospitals. PSCHIATRY.

[23] Nakatani Y (2003). Treatment of mentally disordered offenders. Medical Perspective.*Keiho Zasshi/*. J Criminal law.

[24] Koike J, Kono T, Omachi Y, Murata Y, Kubo M, Kuroki N, Fujii C, Hirabayashi N (2019). Utilization and challenges of the database of the inpatient under Medical Treatment and Supervision Act- Focus Group Interview. Discussion with multidisciplinary staff. Seishinigaku.

[25] Alegría M, NeMoyer A, Falgàs Bagué I, Wang Y、Alvarez K (2018). Social Determinants of Mental Health: where we are and where we need to go. Curr Psychiatry Rep.

[26] Filov GI (2010). Assessment of the criminal recidivism among individuals with mental disorders. Prilozi.

[27] Du J, Huang D, Zhao M, Hser YI (1993). Drug-abusing offenders with co-morbid mental disorders: gender differences in problem severity, treatment participation, and recidivism. Biomed Environ Sci.

[28] Jaffe A, Du J, Huang D, Hser YI (2012). Drug-abusing offenders with co-morbid mental disorders Problem. J Subst Abuse Treat.

[29] Gustavson C, Ståhlberg O, Sjödin AK, Forsman A, Nilsson T, Anckarsäter H (2007). Age at onset of substance abuse a crucial covariate of psychopathic traits and aggression in adult offenders. Psychiatry Res.

[30] Jaffe A, Du J, Huang D, Hser Y (2012). I.Drug-abusing offenders with co-morbid mental disorders: problem severity, treatment participation, and recidivism. J Subst Abuse Treat.

[31] Pollock JM, Mullings JL, Crouch BM (2006). Violent women: finding from the texas women inmates study. J Interpers Violence.

[32] Koike J, Ikeda T, Kuroda O, Koike O, Tsuneoka T, Harima H, Inamoto A, Nakatani (2022). Y.Characteristics of patients with violent recidivism in the forensic mental health system in Japan. J Forensic Psychiatry Psychol.

[33] Baranyi G, Scholl C, Fazel S, Patel V, Priebe S, Mundt A (2019). Severe mental illness and substance use disorders in prisoners in low-income and middle-income countries: a systematic review and meta-analysis of prevalence studies. Lancet Glob Health.

[34] Fazel S, Xenitidis K, Powell J (2008). The prevalence of intellectual disabilities among 12,000 prisoners - a systematic review. Int J Law Psychiatry.

[35] Chester V, Völlm B, Tromans S, Kapugama C, Alexander R. Long-stay patients with and without intellectual disability in forensic psychiatric settings: comparison of characteristics and needs. BJ Psych Open 2018 28;4(4):226–34. 10.1192/bjo.2018.24. 10.1192/bjo.2018.24 PMC6034466 29988967

