# Supplementary material for: Data resource profile of an online database system for forensic mental health services
Source: BMC Med Inform Decis Mak. 2024 Feb 13;24:47. doi: 10.1186/s12911-024-02433-2 (PMC10863232; doi:10.1186/s12911-024-02433-2)
Supplement: Supplementary file 1 — Supplementary Material 1 [file 12911_2024_2433_MOESM1_ESM.docx]

**Online Supplementary**

**Data Resource Profile of an Online Database System for Forensic Mental Health Services**

**Junko Koike*, Toshiaki Kono, Koji Takeda, Yuji Yamada, Chiyo Fujii, Naotsugu Hirabayashi**

**Appendix 1 MTSA Database Major and Broad Category**

| **No** | **Major categories** | **Broad categories** |
| --- | --- | --- |
| 1 | Case No. (Sequential number to identify the case in the file to be submitted. It is not a code unique to the subject, such as a medical record number.) | Basic information |
| 2 | Age at admission, age on the date of data extraction |  |
| 3 | Gender |  |
| 4 | Principal diagnosis and comorbid disorders at the time of the trial decision |  |
| 5 | Principal diagnosis and comorbid disorders after hospitalization in a designated inpatient facilities |  |
| 6 | Index offenses (up to three), classification of victim, etc. |  |
| 7 | Basic information regarding the series of MTSA orders (date of hospitalization, patient identification number unified among all the designated inpatient facilities, re-admission, re-treatment) | Orders and hospitalizations under the MTSA |
| 8 | Information regarding hospitalization to non-regular units in case of bed shortages |  |
| 9 | History of treatment stage transitions |  |
| 10 | Outcomes of inpatient treatment (current treatment status, destination after discharge, reason for transfer, discharge classification, date of admission for each transition stage, and number of days in the hospital and in each stage) |  |
| 11 | Number of referrals to the ethics meeting (long-acting injection, mECT, non-consensual emergency treatment, clozapine prescriptions, number of behavioral restrictions reviewed, number of approvals included, number of reconsiderations included) | Treatment process |
| 12 | Prescription drugs details, drug equivalent conversion amount (at times of data extraction, admission, stage change, 1st and 2nd application for continued hospitalization, and application for discharge) |  |
| 13 | Universal assessment items* ver. 2 or 3  (at times of data extraction, admission, stage change, 1st and 2nd applications for continued hospitalization, and application for discharge) |  |
| 14 | Global Assessment of Functioning (at times of admission, 1st and 2nd applications for continued hospitalization, and application for discharge) |  |
| 15 | International Classification of Functioning, Disability |  |
| 16 | Summary of seclusions and restraints (number of seclusions/restraints, total number of days, elapsed days after admission at the starts of initial and last seclusion and restraints) |  |
| 17 | Details of seclusions and restraints (continuation or termination, elapsed days after admission at start and end) |  |
| 18 | Prefecture where the index offense took place | Basic information |
| 19 | History of juvenile behaviors infringing the criminal code (presence/absence, description (category)) | Criminal and medical treatment history |
| 20 | Criminal history (presence/absence, description (category), the total period of incarceration in the correctional facility, history of hospitalization related to the offence, etc.) |  |
| 21 | Substance use problems (presence/absence, drug used for the first time, primary drug of dependence) |  |
| 22 | Alcohol use problems (presence/absence, description, amount consumed, etc.) |  |
| 23 | Comorbid physical diseases (ICD diagnosis of physical complications) |  |
| 24 | Problematic behaviours in the unit  (interpersonal violence, sexual violence, suicide attempts, number of unauthorized leaves, elapsed days after admission at occurrence, etc.) | Problematic behaviours in the unit. |

*Originally developed risk assessment scale for the MTSA

**Appendix ２ MTSA Database Item Details and Code**

|  | Categories | Subcategories | Classification Code |
| --- | --- | --- | --- |
| 1 | Case No. | Unique patient number across the facilities | 99999999999999 (14 digits) |
|  |  | Code of designated inpatient facility | 1-35 |
| 2 | Age | Age at admission | 999 |
|  |  | Age Classification | 1: under10, 2: 10-19  3: 20-29, 4: 30-39  5: 40-49, 6: 50-59  7: 60-69, 8: 70-79  9: 80-89. 10: over90 |
| 3 | Gender | Gender Classification | 1: male, 2: female |
| 4 | Diagnosis and comorbid disorders at the time of the trial decision | Diagnosis1 | F XXX  (ICD code) |
|  |  | Comorbid disorder1 |  |
|  |  | Comorbid disorder2 |  |
| 5 | diagnosis and comorbid disorders after hospitalization | Diagnosis1 | F XXX  (ICD code) |
|  |  | Comorbid disorder1 |  |
|  |  | Comorbid disorder2 |  |
| 6 | Index offenses | up to three | 0: none, 1: homicide  2: attempted homicide  3: injury  4: rape, 5: attempted rape  6: indecent assault  7: attempted indecent assault  8: robbery  9: attempted robbery  10: arson, 11: attempt arson |
|  | Victim classification | Mother | 0: absence, 1: presence |
|  |  | Father | 0: absence, 1: presence |
|  |  | Sibling | 0: absence, 1: presence |
|  |  | Spouse | 0: absence, 1: presence |
|  |  | Child | 0: absence, 1: presence |
|  |  | Other relatives | 0: absence, 1: presence |
|  |  | Domestic partner | 0: absence, 1: presence |
|  |  | Temporary partner | 0: absence, 1: presence |
|  |  | Acquaintance/Friend | 0: absence, 1: presence |
|  |  | Unknown (person) | 0: absence, 1: presence |
|  |  | Company | 0: absence, 1: presence |
|  |  | Private organization | 0: absence, 1: presence |
|  |  | Public organization | 0: absence, 1: presence |
|  |  | Unknown | 0: absence, 1: presence |
|  |  | Total number of victims | 999 |
| 7 | Basic information regarding the series of MTSA order | Date of hospitalization | 20yy/mm/dd |
|  |  | Treatment order on initial trial | 1: inpatient treatment order  2: outpatient treatment order |
|  |  | Code of designated inpatient facility | 1-35 |
|  |  | Number of readmissions | 999 |
|  | hospital transfer | Code of designated inpatient facility | 1-35 |
|  |  | Reason for hospital transfer | 000000：Blank  000001：Specialized psychiatric treatment (e.g., m-ECT)  000002：Specialized psychiatric treatment completed  000003：Treatment of physical complications  000004：Treatment of physical complications completed  000005：For Discharge coordination (transfer to an institution close to the domicile after discharge)  000006：Others |
|  |  | Number of previous transfers | 999 |
|  |  | Days from admission to transfer | 999 |
| 8 | Information regarding hospitalization to non-regular units in case of bed shortages | Specified medical facility | 0: absence, 1: presence |
|  |  | Days of stay at Specified medical facility | 999 |
|  |  | Status of hospitalization at Specified medical facility | 0: Blank, 1: Continuing  2: Completed |
|  |  | Specified bed | 0: absence, 1: presence |
|  |  | Days of stay at Specified bed | 999 |
|  |  | Status of hospitalization at Specified bed | 0: Blank, 1: Continuing  2: Completed |
| 9 | History of treatment stage transitions | Acute Stage Start Day (Elapsed days from admission) | 999 |
|  |  | Recovery Stage Start Day (Elapsed days from admission) | 999 |
|  |  | Rehabilitation Stage Start Day (Elapsed days from admission) | 999 |
|  |  | Days of Acute stage | 999 |
|  |  | Days of Recovery stage | 999 |
|  |  | Days of Rehabilitation stage | 999 |
| 10 | Outcomes of inpatient treatment | current treatment status | 1: hospitalized  2: transferred  3: discharged  4: others  98: discharged (the order is not clear) |
|  |  | destination after discharge (large classification) | 000000: Blank  000001: Outpatient treatment order  000002: MTSA order terminated  000003: Discharge with protest  000004: Death |
|  |  | destination after discharge (small classification) | 000000: Blank  001001: Outpatient treatment order - Hospitalization  001002: Outpatient treatment order – Residential care facility  001003: Outpatient treatment order - Living with family  001004: Outpatient treatment order – Living alone  002001: MTSA order terminated - Hospitalization  002002 MTSA order terminated – outpatient treatment  002003: MTSA order terminated – no treatment  003001: Discharge with protest  004001: Death (Disease)  004002: Death (Suicide) |
|  |  | number of days in the hospital | 999 |
| 11 | Number of referrals to the ethics meeting (reviewed, approvals included, retentions for reconsideration included) | long-acting injection | 999 |
|  |  | mECT | 999 |
|  |  | non-consensual emergency treatment | 999 |
|  |  | clozapine prescriptions | 999 |
|  |  | behavioral restrictions | 999 |
| 12 | Prescription drugs details, drug equivalent conversion amount | Elapsed days from admission at start | 999 |
|  |  | Item Number | 999 |
|  |  | Rp Number | 999 |
|  |  | Itemization Type | 999 |
|  |  | Drug Code | 999 |
|  |  | Amount | 999 |
|  |  | Unit | 999 |
| 13 | Universal assessment items* ver. 2 or 3  (at times of data extraction, admission, stage change, 1st and 2nd applications for continued hospitalization, and application for discharge) | Psychotic symptoms | 0: none  1: mild  2: Serious |
|  |  | Non-psychotic symptoms |  |
|  |  | Suicide attempts |  |
|  |  | Reflection |  |
|  |  | Life skills |  |
|  |  | Impulse control |  |
|  |  | Empathy |  |
|  |  | Non-sociality |  |
|  |  | Harm of others |  |
|  |  | Personal support |  |
|  |  | Community Factors |  |
|  |  | Stress |  |
|  |  | Substance abuse |  |
|  |  | Practical planning |  |
|  |  | Compliance |  |
|  |  | Treatment Effectiveness |  |
|  |  | Continuity of treatment |  |
| 14 | Global Assessment of Functioning (at times of admission, 1st and 2nd applications for continued hospitalization, and application for discharge) | Score | 999 |
| 15 | International Classification of Functioning, Disability (at times of admission, 1st and 2nd applications for continued hospitalization, and application for discharge) | ensuring one's physical comfort | 999 |
|  |  | managing diet and fitness | 999 |
|  |  | maintaining one's health | 999 |
|  |  | preparing meals | 999 |
|  |  | doing housework | 999 |
|  |  | respect and warmth in relationships | 999 |
|  |  | appreciation in relationships | 999 |
|  |  | tolerance in relationships | 999 |
|  |  | criticism in relationships | 999 |
|  |  | social cues in relationships | 999 |
|  |  | physical contact in relationships | 999 |
|  |  | forming relationships | 999 |
|  |  | terminating relationships | 999 |
|  |  | regulating behaviours within interactions | 999 |
|  |  | interacting according to social rules | 999 |
|  |  | maintaining social space | 999 |
|  |  | managing daily routine | 999 |
|  |  | completing the daily routine | 999 |
|  |  | managing one's own activity level | 999 |
|  |  | handling responsibilities | 999 |
|  |  | handling stress | 999 |
|  |  | handling crisis | 999 |
|  |  | basic economic transactions | 999 |
|  |  | complex economic transactions | 999 |
|  |  | economic self-sufficiency | 999 |
|  |  | products and technology | 999 |
|  |  | natural environment and human-made changes to environment | 999 |
|  |  | support and relationships | 999 |
|  |  | attitudes | 999 |
|  |  | services, systems and policies | 999 |
| 16 | Summary of seclusions and restraints | Number of seclusions | 999 |
|  |  | Days of seclusion | 999 |
|  |  | First seclusion (Elapsed days from admission) | 999 |
|  |  | Latest seclusion (Elapsed days from admission) | 999 |
|  |  | Number of restraints | 999 |
|  |  | Days of restraint | 999 |
|  |  | First restraint (Elapsed days from admission) | 999 |
|  |  | Latest restraint (Elapsed days from admission) | 999 |
| 17 | Details of seclusions and restraints | Code of seclusion or restraint | 1: seclusion  2: restraint |
|  |  | Status of seclusion | 1: Continuing  2: Completed |
|  |  | Status of restraint | 1: Continuing  2: Completed |
|  |  | Elapsed days from admission at start of seclusion | 999 |
|  |  | Elapsed days from admission at start of restraint | 999 |
| 18 | Prefecture where the index offense took place | Code of Prefecture where the index offense occurred | 01-47 |
| 19 | History of juvenile criminal behaviors | Number of criminal offenses | 0: 0,  1:1 time, 2:2-5 times  3: 6-10 times, 4: over 10  99: unknown |
|  | Infringing the criminal code | Homicide | 0: absence, 1: presence |
|  |  | Attempt homicide | 0: absence, 1: presence |
|  |  | Rape | 0: absence, 1: presence |
|  |  | Serious injury | 0: absence, 1: presence |
|  |  | Robby | 0: absence, 1: presence |
|  |  | Motor vehicle theft | 0: absence, 1: presence |
|  |  | Other grand theft | 0: absence, 1: presence |
|  |  | kidnapping | 0: absence, 1: presence |
|  |  | arson | 0: absence, 1: presence |
|  |  | Fraud | 0: absence, 1: presence |
|  |  | drug trafficking | 0: absence, 1: presence |
|  |  | serious violation of traffic laws | 0: absence, 1: presence |
|  |  | escape | 0: absence, 1: presence |
|  |  | Possession of drugs | 0: absence, 1: presence |
|  |  | assault | 0: absence, 1: presence |
|  |  | traffic law violations | 0: absence, 1: presence |
| 20 | Criminal history in adulthood | Maximum progression of criminal justice procedure | 0: No detention,  1: Detention (without indictment),  2: Detention (with indictment)  3: Detention (guilty with indictment)  99: not selected |
|  |  | Homicide | 0: absence, 1: presence |
|  |  | Attempt homicide | 0: absence, 1: presence |
|  |  | Rape | 0: absence, 1: presence |
|  |  | Serious injury | 0: absence, 1: presence |
|  |  | Robby | 0: absence, 1: presence |
|  |  | Theft, robbery, | 0: absence, 1: presence |
|  |  | Drug offenses | 0: absence, 1: presence |
|  |  | Kidnapping | 0: absence, 1: presence |
|  |  | Arson | 0: absence, 1: presence |
|  |  | injury | 0: absence, 1: presence |
|  |  | Sex offense | 0: absence, 1: presence |
|  |  | Negligence offenses | 0: absence, 1: presence |
|  |  | Fraud, forgery, defrauding, deception | 0: absence, 1: presence |
|  |  | Escape, illegal acts | 0: absence, 1: presence |
|  |  | Obstruction of justice, perjury | 0: absence, 1: presence |
|  |  | Various minor crimes | 0: absence, 1: presence |
|  | correctional Institution | The total period of stay in correctional Institution | 0: none, 1: 1-6months  2: 7-12 months  3: 1-2 years, 4: 3-5 years  5: more than 6 years,  99: unspecified/unknown |
|  | Probation History | Details of Probation History | 0: None, 1: Once, 2: Twice  3: Three or more times  99: Unknown |
|  | Parole Compliance Violations | Absence or presence | 0: None, 1: Yes  99: Not selected |
|  | Criminal history―related mental illness | Crime-related hospitalization | 0: absence, 1: presence |
|  |  | (ICD) F0 | 0: absence, 1: presence |
|  |  | (ICD) F1 | 0: absence, 1: presence |
|  |  | (ICD) F2 | 0: absence, 1: presence |
|  |  | (ICD) F3 | 0: absence, 1: presence |
|  |  | (ICD) F4 | 0: absence, 1: presence |
|  |  | (ICD) F5 | 0: absence, 1: presence |
|  |  | (ICD) F6 | 0: absence, 1: presence |
|  |  | (ICD) F7 | 0: absence, 1: presence |
|  |  | (ICD) F8 | 0: absence, 1: presence |
|  |  | (ICD) F9 | 0: absence, 1: presence |
|  |  | ICD code | F XXX |
| 21 | Substance use problems | Presence or absence | 0: absence, 1: present  99: unknown |
|  |  | Drugs used:  amphetamine | 0: absence, 1: present |
|  |  | Drugs used: Cocaine | 0: absence, 1: present |
|  |  | Drugs used: Heroin | 0: absence, 1: present |
|  |  | Drugs used:  Organic solvents | 0: absence, 1: present |
|  |  | Drugs used: Sedatives/Sleeping pills/Painkillers | 0: absence, 1: present |
|  |  | Drugs used: Hallucinogens | 0: absence, 1: present |
|  |  | Drugs used: cannabis | 0: absence, 1: present |
|  |  | Drugs used: others | 0: absence, 1: present |
|  | Age related to drug use | Age of first drug use | 999 or Blank |
|  |  | Age of abuse | 999 or Blank |
|  |  | Age of dependence | 999 or Blank |
|  |  | Age of psychosis | 999 or Blank |
|  |  | Age of related problems | 999 or Blank |
|  |  | Drugs used for the first time | 0: None  1: Methamphetamine  2: Cocaine  3: Heroin  4: Organic solvents  5: Sedatives/sleeping pills/painkillers  6: Hallucinogens  7: Marijuana  8: Other  98: Not selected |
|  |  | primary drug of dependence | 0: None  1: Methamphetamine  2: Cocaine  3: Heroin  4: Organic solvents  5: Sedatives/sleeping pills/painkillers  6: Hallucinogens  7: Marijuana  8: Other  98: Not selected |
| 22 | Alcohol use problems (presence/absence, description, amount consumed, etc.) | Age of first alcohol consumption | 999 or Blank |
|  |  | Age of intoxication | 999 or Blank |
|  |  | Age of physical dependence | 999 or Blank |
|  |  | Age of related problems | 999 or Blank |
|  |  | Age of abuse | 999 or Blank |
|  |  | Age of dependence | 999 or Blank |
|  |  | Age of psychosis | 999 or Blank |
|  |  | Age of initiation of alcohol offenses | 999 or Blank |
|  |  | Duration of consumption (years) | 0: none, 1: up to 2years  2: up to 5 years  3: more than 5 years |
|  |  | Daily consumption (pure alcohol equivalent) | 0: none, 1: less than 40g  2: 40-60g  3: more than 60g  9: unknown |
|  |  | Alcohol use at time of conviction | 0: absence, 1: present  9: unknown |
| 23 | Comorbid physical diseases (ICD diagnosis of physical complications) |  | ICD code |
| 24 | Problematic behaviours in the unit  (interpersonal violence, sexual violence, suicide attempts, number of unauthorized leaves, elapsed days after admission at occurrence, etc.) | count | 999 |
|  |  | Days from admission (1st-10th time) | 999 or Blank |
|  |  | Days from admission  (latest) | 999 or Blank |

*1:’999’ means to fill in the numbers

**Appendix 3 MTSA Database Data Sample**

*1: Some of the items are shown in this figure with dummy values. See the table in Appendix 2 for a comprehensive list of items.
